# Evidence for Startle Effects due to Externally Induced Lower Limb Movements: Implications in Neurorehabilitation

**DOI:** 10.1155/2017/8471546

**Published:** 2017-02-16

**Authors:** Juan M. Castellote, Markus Kofler, Andreas Mayr, Leopold Saltuari

**Affiliations:** ^1^Physical Medicine and Rehabilitation Department, Faculty of Medicine, Universidad Complutense de Madrid and National School of Occupational Medicine, Instituto de Salud Carlos III, Madrid, Spain; ^2^Department of Neurology, Hochzirl Hospital, Zirl, Austria

## Abstract

Passive limb displacement is routinely used to assess muscle tone. If we attempt to quantify muscle stiffness using mechanical devices, it is important to know whether kinematic stimuli are able to trigger startle reactions. Whether kinematic stimuli are able to elicit a startle reflex and to accelerate prepared voluntary movements (StartReact effect) has not been studied extensively to date. Eleven healthy subjects were suspended in an exoskeleton and were exposed to passive left knee flexion (KF) at three intensities, occasionally replaced by fast right KF. Upon perceiving the movement subjects were asked to perform right wrist extension (WE), assessed by extensor carpi radialis (ECR) electromyographic activity. ECR latencies were shortest in fast trials. Startle responses were present in most fast trials, yet being significantly accelerated and larger with right versus left KF, since the former occurred less frequently and thus less expectedly. Startle responses were associated with earlier and larger ECR responses (StartReact effect), with the largest effect again upon right KF. The results provide evidence that kinematic stimuli are able to elicit both startle reflexes and a StartReact effect, which depend on stimulus intensity and anticipation, as well as on the subjects' preparedness to respond.

## 1. Introduction

Displacement of larger body parts, for example, a limb segment, or the whole body, may elicit generalized reactions, for example, postural adjustments, that frequently include muscle responses from upper limbs and neck [1–4]. A sudden unexpected kinematic stimulus while performing other tasks (e.g., walking on a slippery surface) may elicit an undesired motor response, which, if not adequately counteracted, may contribute to a fall [5–9]. In the clinical setting this is of interest as fast passive movements elicited by an examiner or an electromechanical apparatus may trigger both reflexive and voluntary responses [10–17]. If a kinematic stimulus is of sufficient intensity, it may also activate startle reflex circuits, as recently demonstrated in subjects exposed to rear-end impacts, specifically whiplash injuries [18–20]. Startle reactions have most extensively been described in the context of auditory stimulation [21–24] but also with other stimulus modalities such as somatosensory [25–30], or vestibular [31]. Proprioceptive and vestibular pathways conveying impulses generated by postural or kinematic stimuli may evoke startle reflexes or modify them when elicited by other stimulus modalities [32–34]. A so-called “first trial effect,” that is, a larger response to the first as compared to subsequent stimuli, which is typical for auditory startle reactions, has been similarly described for sudden balance disturbances causing vestibular startle reactions [33]. The most widely used indicator muscles for a startle reflex are orbicularis oculi (OOc) [35, 36] and sternocleidomastoid (SCM) [22, 37]. However, unlike all other muscle responses which decline with repeated stimulation owing to habituation [22, 38], OOc activity may persist with repeated acoustic stimuli, suggesting an additional distinct auditory blink reflex [22, 38, 39]. A respective pathway mediating this blink reflex, which involves neurons of inferior colliculus and midbrain reticular formation, has been described in the rabbit [40]. Although SCM responses may be absent in a substantial percentage of startle trials [41] they still seem to be the last proper startle reflex responses to disappear due to habituation, unlike responses in OOc.

Startle reflexes habituate less when the subject is prepared to perform a voluntary movement [39, 42]. Unexpected presentation of a startling stimulus in a reaction time (RT) paradigm simultaneous to the imperative signal (IS) results in a startle reflex and an early release of a preplanned volitional motor program. This phenomenon is called StartReact effect [39, 42] and has to date been most extensively explored with acoustic stimuli, but also with other modalities including vestibular and kinematic stimuli [34, 43–45].

Notably, an accelerated release of a motor response may also be directly related to the intensity of the IS [46–49] and may thus erroneously be mistaken as a StartReact effect [41, 50]. Concomitant responses in OOc and/or SCM, indicating a generalized startle reaction, may serve to differentiate early released motor responses due to stimulus intensity alone from those due to additional startle effects. Furthermore, a careful use or avoidance of low-intensity prepulses, which are known to suppress startle responses while preserving the StartReact effect [51], may help to further characterize the observed responses [52].

Startle reflexes and StartReact effect are of clinical interest in both diagnostic and therapeutic facets of neurorehabilitation. (1) Startle reflexes could potentially “contaminate” the measurement and quantification of muscle stiffness with robotic exoskeletons in patients affected by spasticity. (2) Startling stimuli may serve to trigger prepared actions, for example, reaching and grasping, in patients suffering from stroke, whose corticospinal tract is damaged and thus incapable of conveying the necessary neural impulses for mediating the requested movements [53–55]. (3) Novel therapeutic and preventive strategies could implement startling stimuli for subjects to learn quick motor reactions counteracting disturbances which might cause falls; main target groups include elderly, frail, and handicapped persons and in particular those exposed to unstable or slippery surfaces. (4) Finally, the study of kinematic stimuli used as a trigger of startle responses is of physiological interest, as this kind of stimuli is long-lasting and thus differs considerably from brief auditory stimuli which are usually used to elicit startle responses. Recent studies have suggested that kinematic stimuli may indeed elicit a StartReact effect, however, without substantiating recordings obtained from SCM or OOc [56, 57]. Furthermore, responses were recorded in those muscles where kinematic stimuli were applied; thus voluntary responses, long loop reflexes (LLRs), and possibly startle reflexes may have overlapped. Finally, it was not possible to unequivocally differentiate whether the short latencies of the obtained responses were due to kinematic stimulus intensity or due to startling effects. Similarly, Ravichandran et al. [34] studied response modification by auditory stimuli or movement perturbation during the LLR time window, again only in the limb being moved. Hence, both voluntary and reflex responses were concomitantly evaluated in the same muscles. These authors also recorded the presence of SCM activity and concluded that there was a startle effect only if a subject was prepared to react with a preprogrammed movement. However, the requirement of a movement plan to elicit a startle reaction is not absolutely mandatory. Although it is known that habituation of startle responses is significantly reduced in RT paradigms [58], extensive research has also shown startle-related SCM or OOc activity in subjects in the absence of any required movement [22, 59, 60]. Additionally, SCM activity can be part of a postural reaction or part of the motor pattern of performing a forceful and fast elbow extension rather than being part of a startle response. Recently, Campbell et al. [43] have reported shorter RTs and a StartReact effect with stimuli evoking combined kinematic and vestibular influences. In order to separate out startle reflex signs in SCM from proprioceptive or vestibular responses, or from muscle activity inherent to a particular motor program, kinematic or postural stimuli should avoid head movements. More recently Forgaard et al. [61] also observed voluntary response modifications following limb perturbation during the LLR time window. In a separate experiment, they also recorded from SCM and OOc in three subjects, in whom occasional startling auditory stimuli were presented together with limb perturbation. The authors concluded that startle signs in SCM and OOc following auditory stimuli were present only when subjects were prepared to move. However, as limb perturbation was present in all trials, these kinematic stimuli were expected, whereas additional auditory stimuli were unexpected. Startle-related activity, however, may easily appear in the absence of prepared movements when stimuli are sufficiently surprising [22, 59, 60]. Thus, if limb perturbation had been unexpected, it might in fact have evoked a startle reaction.

With these premises in mind, we were interested in further exploring whether incremental kinematic stimuli in the lower limbs are able to shorten RTs in the upper limbs and whether they are able to elicit a startle reflex and a StartReact effect. Specifically, we wanted to separate voluntary responses from stretch reflexes, and therefore we used different limbs for stimulation and recording. In order to document the presence of a startle reflex, we recorded from OOc and SCM. Responses in either muscle should help differentiating whether an accelerated voluntary response is due to the strength of the stimulus or due to a superimposed StartReact effect. Recording of OOc and SCM activity when performing the requested upper limb movement in response to expected kinematic stimuli in the lower limbs aimed to confirm that the applied stimulus-response protocol excludes OOc or SCM activity as part of the motor task, or as a part of a postural reaction (i.e., head stabilization), which, if present, could be erroneously mistaken as expression of a startle reflex. Prepulses, which are known to suppress startle responses in OOc and SCM while preserving the StartReact effect, were included in some trials with unexpected high intensity stimuli, in order to compare these responses with those without prepulse, and to confirm the startling value of the delivered stimulus.

## 2. Material and Methods

### 2.1. Ethical Approval

Eleven healthy subjects (9 females, 2 males, age 29–51 years) took part in the experiments. All were self-reported right-handers with normal or corrected-to-normal vision and were free from any neurological deficit that could affect the execution of the task. Subjects gave their written informed consent for the experiment in accordance with the Declaration of Helsinki, which was approved by the local Institutional Review Board.

### 2.2. Setup

Subjects were placed in a driven electromechanical gait robot, Lokomat (Hocoma, Switzerland), with each lower limb strapped to an exoskeleton, adjusted to individual anthropometric measures. The Lokomat produced passive knee joint movements at various angular velocities. Subjects were suspended in the air during trial periods by a harness around the torso attached to an over-head Body Weight Support System (with deflection pulleys). Handrails on either side of the subject at waist level allowed supporting with the hands if necessary. A band strapped between both bars in front of the subject enabled resting the forearms. A possible startle reaction was monitored by recording surface electromyographic (EMG) activity from the right OOc and SCM. Surface EMG related to wrist extension (WE) was recorded from right extensor carpi radialis muscle (ECR). Single sweeps of 4 s were recorded, including a 900 ms prestimulus delay using routine electrodiagnostic equipment (Viking IV, Nicolet Biomedical, Madison, Wisconsin). Filter settings were 10–10000 Hz. Electrical constant voltage stimuli of 0.1 ms duration and 1.5 times perception threshold were generated with a Digitimer D180A. These stimuli served as prepulse and were only occasionally delivered in few trials through ring electrodes placed on the left index finger, 100 ms preceding KF. Both Lokomat and electrodiagnostic system were synchronized, with the sweep being triggered by the Lokomat as soon as a KF of 80° was initiated from a starting point with completely extended knees, irrespective of induced angular velocity. Subsequent knee extension back to the starting point was always performed at a low angular peak velocity (less than 10°/s).

### 2.3. Procedure and Test Sequence

Subjects were informed that they were going to be suspended in the Lokomat and that there would be a series of trials and that unless otherwise advised they should perform a fast WE (simple RT paradigm) as soon as they perceived the IS, that is, perception of passive KF induced by the robot. They were told that if they felt uncomfortable due to the maintained position they should tell the experimenters immediately, so that they would be lowered to standing on the floor, or, if desired, would be detached from the system. The study included also established stops and lowering of the subjects from the electromechanical device at different times. The study contained a predetermined workflow of trials. A trial included a verbal warning signal for the subject to be prepared “ready!”, the IS delivered at a variable latency of 1 to 3 s following the warning signal, and recording of the subject's responses. The experimental session was composed of three consecutive blocks of trials. Within each block, trials were separated by a minimum of 45 seconds, time required by the Lokomat system to again reach the starting position, to provide sufficient time for subjects to achieve a comparable resting condition, and to avoid influence of one trial upon the subsequent one.

In a pilot test, one subject was randomly exposed to different passive left KFs reaching peak velocities between 6°/s and 240°/s, which were all clearly perceived. The fastest velocity was previously used for measurements of muscle stiffness in patients with upper motoneuron lesions [13]. In this pilot subject, occasional startle signs were present only at 240°/s, but not with lower angular peak velocities. These findings were used to design a fixed experimental protocol of trials for the remaining subjects, applying three preestablished peak velocities, 6°/s, 60°/s, and 240°/s, for which the system required 11.4, 3.2, and 1.2 s, respectively, to reach the determined velocity within a maximum 80° movement range.

In block 1, at the beginning of each experimental session, low intensity tone bursts (60 dB nHL, 500 Hz, 10 ms duration) were used as IS. Subjects were instructed to perform only a fast WE upon hearing the tone (condition “WE-only”). Five WE-only trials were repeated in order to accustom the subject to the suspension in the Lokomat system and to depict the subject's movement pattern employed when briskly raising their hand, specifically to check whether there was any EMG activity in SCM associated with the task, for example, anticipatory postural adjustments, which could interfere with analysis of responses in the subsequent blocks of trials.

In block 2, subjects were instructed to respond with a fast WE upon perceiving the IS, from that point on passive KF. Different angular velocities of left KF interspersed with occasional fast right KF were applied in pseudorandom order, without informing subjects beforehand about the type of upcoming stimuli. Accordingly, based on velocity and presence/absence of startle signs, resulting recordings of each trial were post hoc grouped and categorized for analysis into different “conditions.” The type of trials and resulting conditions were as follows:

- Trials at 240°/s passive left KF, resulting in conditions “240React” and “240StartReact”
- Trials at 60°/s passive left KF, resulting in conditions “60React” and “60StartReact”
- Trials at 6°/s passive left KF, resulting in condition “6React”; none of these trials showed any startle signs; hence there was no condition “6StartReact”
- Trials at 240°/s passive left KF, in which subjects received the prepulse in addition to passive KF, resulting in conditions “240PrepReact” and “240PrepStartReact”
- Trials at 240°/s passive KF, in which the contralateral (right) leg was moved as IS, resulting in conditions “240ContraReact” and “240ContraStartReact”; in order to render these stimuli less expected, these trials were only introduced during the last fourth of the experimental block; as subjects were previously continuously exposed to left leg movements, they were supposedly less prepared to anticipate their right leg to be moved

Each of the 240°/s trials was interspersed with at least 5 trials at 6°/s and occasional trials at 60°/s, in order to avoid rapid habituation. For each condition, OOc and SCM activity was visually checked online in order to obtain a sufficient number of recordings containing startle signs, that is, at least three 240StartReact, two 240React, and two 240ContraStartReact recordings per subject. Thus, at least 40 trials at lower angular velocities were delivered per subject. If a subject generated too few overt startle responses in any given condition, additional trials were added at 240°/s, each interspersed with at least 5 trials at lower angular velocities. Thus, the total number of trials varied across subjects as it was not known beforehand how many trials at 240°/s eventually had to be repeated in order to achieve the required number of startle responses per condition.

At the end of this block, subjects were exposed to a subset of left KF trials at 240°/s, in which they were asked to remain relaxed and specifically not to perform WE. These trials were interspersed with some trials at 6°/s and 60°/s in order to keep subjects alert but uncertain about the velocity of the upcoming IS. This subset of trials at 240°/s was analyzed specifically to rule out EMG activity in SCM as part of postural adjustment to the leg displacement and served as controls for other trials at the same velocity. The resulting conditions were “240Control” and “240StartControl.”

Finally, block 3 was comprised of five trials in which subjects were explicitly informed about both side and velocity of the forthcoming leg movement (left leg, 240°/s) and in which they were asked to respond with fast WE. Resulting recordings were categorized accordingly into conditions “240Known” and “240StartKnown.”

The resulting number of trials per condition and per subject, which were analyzed, is depicted in Table 1.

### 2.4. Data Processing and Analysis

EMG activity was full-wave rectified before analysis. For each subject and condition, EMG characteristics were determined separately for the selected muscles, and median values were used for statistical inferences. SPSS 22.0 was used for statistical analysis. Unless otherwise specified, data are shown as median and 95% confidence intervals (in brackets). OOc and SCM activity was accepted to represent a startle reflex, if it appeared at an appropriate latency, lasted more than 50 ms, and exceeded two standard deviations of baseline activity established during a 200 ms time window preceding IS (i.e., movement onset induced by the Lokomat).

Startle reflex onset latency was determined by visual inspection of the respective traces from 40 to 100 ms for OOc and from 50 to 120 ms for SCM following IS. These time windows were used in accordance with previous reports [22, 60, 62] and taking into account differences in afferent conduction time for a passively moved leg as compared to a short auditory stimulus. Due to occasional difficulty in determining exact reflex response durations, the magnitude of startle reflexes was expressed for each muscle as the EMG area-under-the-curve (iEMG) during a predefined 50 ms segment starting at response onset.

For WE, EMG onset latency in ECR was determined by visual inspection in a time window with an upper limit at 500 ms following IS for trials at 60°/s and 240°/s and 2000 ms for trials at 6°/s, as estimated from the pilot subject. Responses were accepted when EMG activity lasted more than 100 ms and exceeded two standard deviations of a 200 ms prestimulus baseline. ECR response magnitude was calculated as iEMG during a 100 ms window following response onset, as respective muscle bursts were usually longer-lasting than those obtained in OOc and SCM. For statistical inference purposes, iEMG values were normalized for each subject relative to individual maximum voluntary activity. Median values and 95% confidence intervals of EMG onset latencies and iEMG were calculated for all subjects and conditions.

Percentages of trials with startle signs were calculated separately for each subject and type of trial and were then compared among trial types using Friedman *χ*^2^ test. The distribution of startle signs in OOc and SCM was compared between conditions 240StartReact and 240ContraStartReact using Wilcoxon signed rank test.

Friedman *χ*^2^ test was also applied to compare the effect of stimulus intensity (among conditions 6React, 60React, and 240React), of prepulse stimulation (among conditions 240React, 240StartReact, and 240PrepStartReact) and of preparedness in regard to the leg to be moved (expected left leg: conditions 240React and 240StartReact; unexpected right leg: conditions 240ContraReact and 240ContraStartReact), on latencies and iEMG of ECR responses, respectively. Pairwise post hoc comparisons between categorized conditions were performed using Wilcoxon signed rank test for latencies and iEMG of ECR responses in trials containing WE. Wilcoxon signed rank test was also used to compare the effect of presence or absence of startle signs on latencies and iEMG of ECR responses in all trial types employing 240°/s velocity. Startle response latencies and iEMG in OOc were compared between conditions 240StartReact and 240ContraStartReact applying Mann—Whitney *U* test, as not all subjects presented with startle signs in OOc in both conditions, precluding pairwise comparisons. Startle responses in SCM in these two conditions were compared with Wilcoxon signed rank test. The level of significance was set at *P* < 0.05, which was adjusted for multiple comparisons using Bonferroni correction. Effect size was calculated with Cohen's* d*.

## 3. Results

All subjects performed the study without difficulty. Two subjects asked for intermittent release from suspension in order to stand on their feet for a short period of time during the experiment. In order to acquire a sufficient number of trials per condition at 240°/s containing startle signs, trials of different velocities occasionally had to be repeated according to protocol, thereby amounting to 65 to 85 trials per subject.

### 3.1. Effects of Kinematic Stimulus Intensity on Reaction Time

In response to passive KF at different angular velocities, used as IS, all subjects performed the required WE. Stimulus intensity had a significant effect on ECR latencies, when comparing conditions 6React, 60React, and 240React (Friedman *χ*^2^ = 20.18, df = 2, *P* < 0.0001). Latencies of ECR responses were longer for 6React [1392 (1184, 1516) ms] than for 60React [324 (282, 412) ms] and were shortest for 240React [268 (224, 320) ms]. Pairwise post hoc comparisons showed significant differences between 6React and 60React (*Z* = −2.9, *P* < 0.01; Cohen's *d* = 9), 6React and 240React (*Z* = −2.9, *P* < 0.01; Cohen's *d* = 11), and 60React and 240React (*Z* = −2.8, *P* < 0.01; Cohen's *d* = 1). Burst size in ECR, expressed as iEMG, was smaller for 6React [244 (167, 462) *µ*V*∗*ms] than for 60React [370 (323, 528) *µ*V*∗*ms] and largest for 240React [517 (306, 702) *µ*V*∗*ms]. There were significant overall differences (Friedman *χ*^2^ = 17.63 df = 2, *P* < 0.001), with pairwise post hoc differences between 6React and 60React (*Z* = −2.9, *P* < 0.016; Cohen's *d* = 1) and between 6React and 240React (*Z* = −2.9, *P* < 0.016; Cohen's *d* = 2) but not between 60React and 240React (*Z* = −1.9, *P* = 0.05; Cohen's *d* = 1).

### 3.2. Startle Reflexes due to Kinematic Stimuli

In WE-only condition, there was no evidence of SCM activity before WE, documenting no activity associated with head displacements due to brisk wrist movement and/or anticipatory postural adjustments. Condition 240Control (in which subjects were asked not to perform WE) also revealed no associated SCM activity. Thereby we excluded the possibility that SCM activity may appear as part of a postural reaction to passive KF.

**Figure 1:**
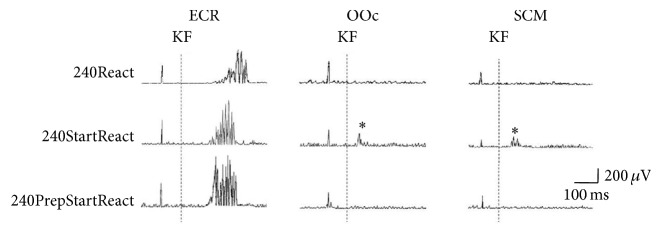
Representative trials from one subject depicting responses from extensor carpi radialis (ECR), orbicularis oculi (OOc), and sternocleidomastoid (SCM) muscles in conditions 240React, 240StartReact, and 240PrepStartReact. The traces show a leftward displacement (anticipation) and larger integrated EMG activity in ECR in the 240StartReact and 240PrepStartReact trials as compared to the 240React trial. Startle-related responses in OOc and SCM (marked with asterisks: *∗*) are present only in the 240StartReact trial, as they are suppressed by the prepulse in the 240PrepStartReact trial. KF: start of knee flexion. The vertical deflection 100 ms before KF is a stimulus artifact.

**Figure 2:**
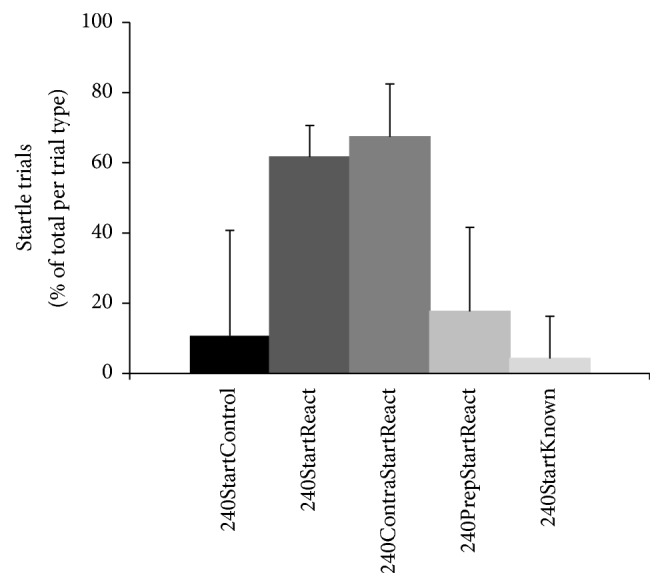
Amount of trials per trial type at 240°/s containing startle signs. For each of the eleven subjects the percentage of trials per trial type at 240°/s containing startle signs in orbicularis oculi or sternocleidomastoid muscles was calculated relative to the total number of respective trials (e.g., number of 240StartReact trials divided by [number of 240StartReact trials plus number of 240React trials] times 100). For descriptive purposes means + standard deviation of these percentages are shown. Note the highest “yield” of startle responses in conditions 240ContraStartReact and 240StartReact, with lower mean percentages in conditions 240PrepStartReact, 240StartControl, and 240StartKnown.

There were no startle signs in OOc or SCM at all in trials at 6°/s and only few in trials at 60°/s (0-1/subject). In contrast, at 240°/s startle indicators were present in 45% of trials.  Figure 1 depicts a representative example at 240°/s. The number of trials at 240°/s containing startle signs in OOc or SCM (Table 1) depended on the type of trial (see Methods) with significant differences among trial types (Friedman *χ*^2^ = 32.28, df = 4, *P* < 0.0001) (Figure 2). Post hoc testing revealed a higher number of trials with startle signs for 240°/s left KF (which resulted in conditions 240React and 240StartReact) and 240°/s right KF (240ContraReact and 240ContraStartReact) than for those with prepulse (240PrepReact and 240PrepStartReact) (*Z* = −2.9, *P* < 0.007 for both comparisons; Cohen's *d* = 3 for both) or those without WE (240Control and 240StartControl) (*Z* = −2.7, *P* < 0.007; *Z* = −2.6, and *P* < 0.007, resp.; Cohen's *d* = 2 for both), or those in which subjects knew side and velocity of KF (240Known and 240StartKnown) (*Z* = −2.9, *P* < 0.007 for both comparisons, Cohen's *d* = 6 for comparison with 240°/s left KF, and Cohen's *d* = 4 for comparison with 240°/s right KF). The amount of trials with startle signs did not differ significantly between 240°/s left KF (240React and 240StartReact) and 240°/s right KF (240ContraReact and 240ContraStartReact) (*Z* = −0.8, *P* = 0.4; Cohen's *d* = 0.4).

**Figure 3:**
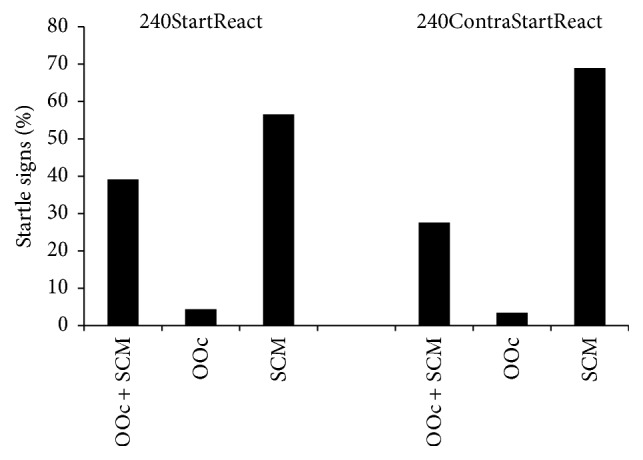
Presence of startle signs according to stimulus location, obtained in eleven subjects and calculated as described in Figure 2. The probability of startle signs was similar for trials in which the most frequently moved (left) leg was used as imperative signal, as compared to those trials in which the less expected (right) leg was used (conditions 240StartReact and 240ContraStartReact, resp.). For descriptive purposes values in orbicularis oculi (OOc), sternocleidomastoid (SCM), or both muscles are represented as mean percentage of trials across all subjects.

As startle signs were absent in trials at 6°/s and scarce in 60°/s and in some 240°/s conditions (i.e., 240PrepStartReact, 240StartControl, and 240StartKnown), subsequent statistical comparisons were only performed between conditions 240StartReact and 240ContraStartReact. The distribution of startle signs in OOc and SCM was similar in both conditions (for OOc: *Z* = −0.1 and *P* = 0.9; Cohen's *d* = 0.1; for SCM: *Z* = −0.4 and *P* = 0.6; Cohen's *d* = 0.2), that is, irrespective of the leg being moved (Figure 3). In both conditions combined, startle signs were either found in OOc (4%), SCM (61%), or both (35%).

**Figure 4:**
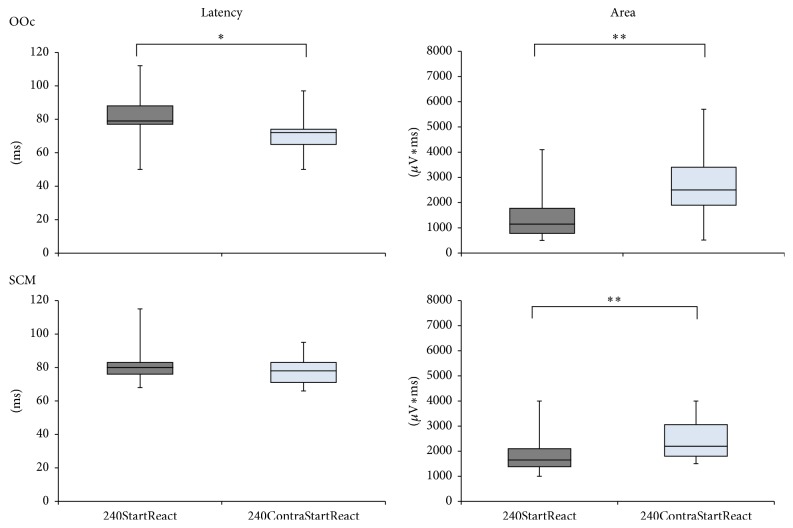
Latency and area-under-the-curve of startle reflexes according to stimulus location. The plots represent onset latencies and iEMG in orbicularis oculi (OOc) and sternocleidomastoid (SMC) muscles from all subjects obtained in conditions 240StartReact and 240ContraStartReact. Each box represents 50% of all values intersected by the median; the whiskers indicate the smallest and largest values. When the imperative signal was applied in the less expected (right) leg (condition 240ContraStartReact), orbicularis oculi (OOc) latencies were significantly shorter than when imperative signal was applied in the most frequently moved (left) leg (condition 240StartReact). In condition 240ContraStartReact both OOc and SCM showed significantly larger responses. Asterisks above the boxes define the level of significance of between-group comparisons (^*∗*^*P* < 0.05; ^*∗∗*^*P* < 0.01).

In OOc, startle latency was significantly shorter in condition 240ContraStartReact [72 (50, 74) ms] than in 240StartReact [79 (77, 112) ms] (*Z* = −2.1, *P* < 0.05; Cohen's *d* = 0.2) but not in SCM [240ContraStartReact: 78 (69, 87) ms; 240StartReact: 80 (75, 85) ms; *Z* = −0.9, *P* = 0.3; Cohen's *d* = 0.5]. Startle burst size (iEMG) was significantly larger in condition 240ContraStartReact as compared to 240StartReact in both muscles [OOc: 2500 (525, 5600) versus 1150 (500, 6075) *µ*V*∗*ms and *Z* = −2.6, *P* < 0.01, Cohen's *d* = 0.5; SCM: 2200 (1590, 3800) versus 1650 (1350, 3500) *µ*V*∗*ms and *Z* = −2.8, *P* < 0.01; Cohen's *d* = 0.5] (Figure 4).

### 3.3. Effects of Startle on Reaction Time (StartReact Effect)

**Figure 5:**
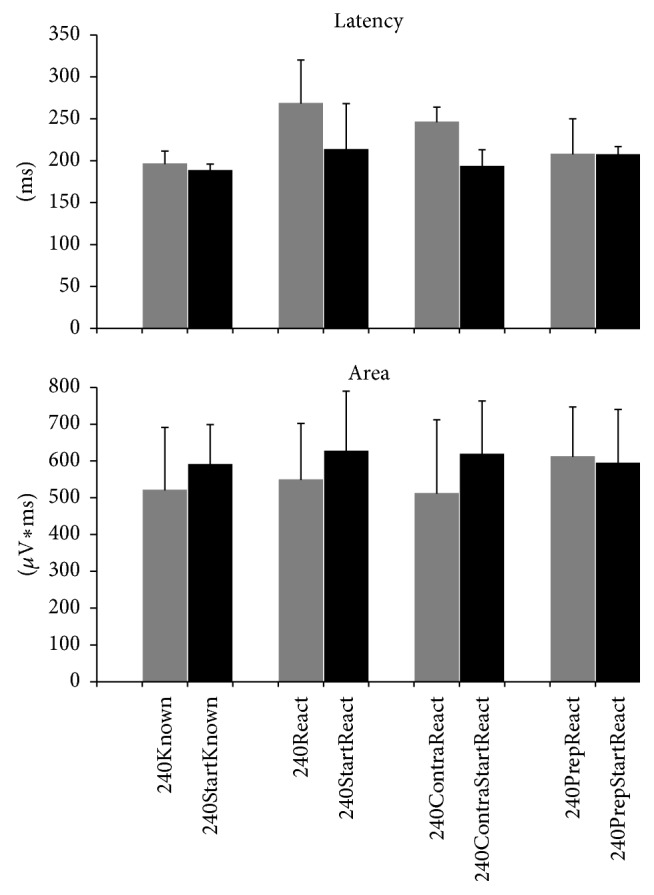
StartReact effects according to condition at 240°/s. Trials containing startle signs (conditions depicted in black) showed shorter latencies and larger responses in extensor carpi radialis (ECR) electromyographic recordings than those without startle signs (corresponding conditions depicted in grey), except for those with prepulse stimulation (data are medians and confidence intervals). Due to study protocol requirements, however, only few trials included a prepulse and, as expected, almost none contained startle signs (condition 240PrepStartReact), while most of them did not contain startle signs (condition 240PrepReact). Both showed ECR latencies and amplitudes similar to other conditions with startle signs, indicating that trials with a prepulse showed a StartReact effect, irrespective of the presence or absence of overt startle signs in orbicularis oculi or sternocleidomastoid muscles.

In 240°/s trials with WE, latencies of ECR responses were significantly shorter in conditions with startle signs (240StartReact, 240PrepStartReact, 240ContraStartReact, and 240StartKnown, all combined) [210 (192, 254) ms] than without startle signs (240React, 240PrepReact, 240ContraReact, and 240Known, all combined) [233 (207, 249) ms] (*Z* = 2.25 and *P* < 0.05; Cohen's *d* = 0.05), suggesting a StartReact effect. ECR iEMG was larger in those conditions combined with startle signs [599 (546, 674) *µ*V*∗*ms] than in those without startle signs [548 (496, 620) *µ*V*∗*ms] (*Z* = 3.46 and *P* < 0.001; Cohen's *d* = 0.3), also in agreement with a StartReact effect (Figure 5). No inferences were drawn for all as a group, as conditions 240StartKnown and 240PrepStartReact contained so few startle responses (0-1/subject) that they and their corresponding counterparts (240Known and 240PrepReact) were excluded from further comparisons.

In order to document a StartReact effect, that is, acceleration of a prepared movement (here: WE) by a startling stimulus and to show the influence of preparedness (here: left KF, “expected side” versus right KF “unexpected side”) we compared latencies and iEMG in ECR responses applying Friedman *χ*^2^ test in conditions 240React, 240StartReact, 240ContraReact, and 240ContraStartReact. ECR latencies were significantly different among conditions (Friedman *χ*^2^ = 16.74, df = 3, *P* < 0.001), being shorter in conditions containing startle signs [203 (169; 240) ms] than in those without startle signs [257 (215, 292) ms]. ECR latencies were also shorter in trials with right “unexpected” KF [219 (186, 238) ms] versus left “expected” KF [240 (197, 294) ms]. Pairwise post hoc comparisons showed that 240StartReact latencies were significantly shorter than 240React latencies (*Z* = 2.9 and *P* < 0.01; Cohen's *d* = 0.9) and that 240ContraStartReact were significantly shorter than 240ContraReact latencies (*Z* = 2.9, *P* < 0.01; Cohen's *d* = 1). There were no significant differences between 240React and 240ContraReact conditions (*Z* = 1.4, *P* = 0.1; Cohen's *d* = 0.6), nor between 240StartReact and 240ContraStartReact conditions (*Z* = 1.6 and *P* = 0.1; Cohen's *d* = 0.6). ECR iEMG was also significantly different among conditions (Friedman *χ*^2^ = 10.21, df = 3, *P* < 0.05), being larger in conditions with startle signs [621 (422, 796) *µ*V*∗*ms] than without [514 (359, 707) *µ*V*∗*ms], but similar in trials with right “unexpected” KF [564 (479, 737) *µ*V*∗*ms] versus left “expected” KF [571 (302, 766) *µ*V*∗*ms]. Pairwise post hoc comparisons showed significantly larger iEMG values in 240StartReact versus 240React (*Z* = 2.6, *P* < 0.01; Cohen's *d* = 1) and in 240ContraStartReact versus 240ContraReact (*Z* = 2.9, *P* < 0.01; Cohen's *d* = 1). There were no significant differences between 240React and 240ContraReact conditions (*Z* = 0.4, *P* = 0.7; Cohen's *d* = 0.6) and between 240StartReact and 240ContraStartReact conditions (*Z* = 0.8, *P* = 0.4; Cohen's *d* = 0.7). ECR latency and iEMG values for each condition are depicted in Figure 5.

The effect of a prepulse on WE was assessed in order to confirm the presence of a StartReact effect. ECR latency was 207 (171, 250) ms in 240PrepReact, which was close to values obtained in conditions containing startle signs. There was a significant difference in ECR latency among 240React, 240StartReact, and 240PrepReact (Friedman *χ*^2^ = 13.8, df = 2, *P* < 0.001). Post hoc test showed that the differences were between 240React and 240StartReact (*Z* = 2.9, *P* < 0.016; Cohen's *d* = 0.9) and between 240React and 240PrepReact (*Z* = 2.6, *P* < 0.016; Cohen's *d* = 1). ECR iEMG was 611 (231, 746) *µ*V*∗*ms in 240PrepReact, which did not differ significantly from 240React and 240StartReact (Friedman *χ*^2^ = 5.6, df = 2, *P* = 0.06).

## 4. Discussion

In the present study different intensities of a kinematic stimulus modified RT responses. The highest intensity resulted in shortest RT and largest response magnitude. Kinematic stimuli exceeding a certain intensity level, and particularly when unexpected, may themselves evoke startle reflexes. They also generate a StartReact effect, as explored by a voluntary response task in a muscle distant from stimulus location. Additionally, the magnitude of the StartReact effect is inversely related to the degree of preparedness of a subject to receive the stimulus.

### 4.1. Effects of Stimulus Intensity on Reaction Time

Shorter RTs and larger response sizes associated with stronger stimuli concur with previous reports in other domains [41, 46, 47]. However, latency measurements of reactions to kinematic stimuli deserve further discussion. For modalities employing very brief stimuli, such as an electrical pulse or an acoustic click, it seems clear that the beginning of stimulus rise time equals stimulus onset, thus being the reference point for response latency measurements. But for long-lasting stimuli, such as radiant or contact heat [63–65], or movements (e.g., present study), the reference point in time is not so evident. Yet, long-lasting stimuli are of special interest as they occur in daily life (e.g., slippery or unstable surfaces and pushes). So far several attempts have been made to describe trigger characteristics in kinematic studies (e.g., pendulum test, isokinetic assessments) using movement as a trigger for reflexes [66, 67] or assessing muscle tone, spasticity, or rigidity [66–68]. However, until a precise time delimitation of the trigger can be established, movement onset seems to be the most plausible reference point for determining latencies following kinematic stimuli. In the present study, peak velocity of the Lokomat was reached around 250 ms after movement initiation in the fastest trials (240°/s). As RTs were in the same range, peak velocity does not seem to trigger the responses. Instead, very early changes in leg position may have served as trigger. The RT of 268 ms in ECR in the present study concurs well with previous reports: for example, single RTs of 210 ms [69] and 225 ms [51], respectively, for EMG responses in ECR following a visual IS; 220 ms for pushing a handheld microswitch button following an auditory IS, and 250 ms following a visual IS, in a task applying postural perturbations [70]. Longer RTs following limb displacement can easily be explained by the nature of kinematic stimuli, being longer-lasting than auditory or visual stimuli, and thus reaching “subjective perceptional threshold” relatively late.

### 4.2. Startle Reflexes due to Kinematic Stimuli

The movement applied with the Lokomat was able to elicit a startle reflex on some occasions. This was mainly the case in trials with the highest intensity, although not in all conditions. As with other kinds of stimuli, a certain level of intensity and unexpectedness were prerequisite to elicit a startle reflex [35, 41, 71]. Startle latencies were not as short as described with acoustic stimulation [22, 72] likely due to both differences in afferent conduction times and kinematic stimulus characteristics as described above. Yet kinematic stimuli proved to be indeed capable of eliciting startle reflexes on their own, but the longer latencies need to be taken into account when exploring startle reflexes and StartReact effects related to postural perturbations.

We designed a protocol that included few trials at the highest intensity among a majority of trials at lower intensities in one (left) leg. Thereby we were able to obtain startle reflexes in OOc and SCM, possibly related to some degree of a subject's surprise (240StartReact). Most startle responses were present in SCM, possibly due to an additional influence of posture (keeping the head upright in a suspended situation) [73]. The intended addition of a voluntary task in the experimental setup aimed to reduce the rate of habituation [74, 75] and may thus have facilitated the presence of startle reflexes in either muscle, even until the end of the experiment [39, 51, 59]. When no WE was required, few startle reflexes were observed, possibly due to a lower level of readiness or a higher rate of habituation. When WE was required, the occasional lack of startle reflexes in some trials with the highest intensity may as well either be due to habituation [33, 39] or due to a subject's temporarily reduced readiness to perform the required task, as described also with other stimulus modalities [33, 74, 75].

Subjects were not explicitly informed that trials would occasionally be interspersed in the other (right) leg during the last fourth of the session. Thereby we were able to obtain largest startle reflexes with shortest latencies in OOc and SCM (240ContraStartReact), possibly related to the highest degree of surprise as the stimulus appeared rather unexpectedly in the leg contralateral to the one which was displaced in the majority of trials throughout the experiment. Consequently, kinematically induced startle reflexes may show EMG responses of different magnitude depending on the level of attention, as previously described in other domains [76–78].

The present study design is set out to overcome some limitations of previous studies, which have explored kinematic stimuli without specifically recording from startle reflex indicator muscles [56, 57], or applying expected and thus possibly not sufficiently surprising stimuli [61], or without providing evidence of absence of postural reactions in SCM associated with the requested voluntary arm movement which was intentionally disturbed [34]. Furthermore, all these studies explored motor responses in the same passively displaced limb during the time window of LLR [34, 56, 57, 61]. Hence it was difficult to ascertain the suspected startling nature of kinematic stimuli, and, if startle signs were present in OOc or SCM, the superimposition of reflexive or voluntary responses in the limb being moved made a clear characterization of startle responses in the same extremity muscles difficult.

To rule out that the obtained SCM activity was due to reactive head stabilization as part of postural adjustment related to brisk WE movements [79, 80] or that it was part of the motor program for fast WE to be executed with strong effort [79, 80], we included trials containing just WE and no other task. SCM activity may also appear related to lower limb movement. Finally, SCM activation may be due to startle evoked by movement-related vestibular influence, as previously reported [33, 81]. Therefore, we included trials containing just leg movement with no WE. In these specific experiments, however, we observed no SCM activity, suggesting that the SCM activity observed in other trials was indeed startle-related.

In order to further confirm that the observed responses in OOc and SCM were indeed startle reflexes, we included some trials containing prepulse stimuli. Prepulses are known to inhibit startle reflexes but at the same time to maintain the StartReact effect [51, 59]. Indeed, trials with a prepulse showed an accelerated WE even in the absence of startle reflex signs in OOc and SCM, in agreement with a StartReact effect in WE and concomitant profound startle reflex suppression in OOc and SCM.

Considering all factors described above, we conclude that the kinematic stimuli applied at the highest intensity were indeed able to elicit startle reflexes.

### 4.3. Effects of Startle Reflex on Reaction Time (StartReact Effect)

All subjects performed WE in all trials when required, indicating that also stimuli of lowest intensity were able to trigger these responses. Latency variability was highest for the lowest intensity, consistent with the subjects' difficulty in discriminating these stimuli [82]. In trials at the highest intensity, response latencies were shorter and muscle burst activity was larger when startle reflex signs were present, as previously shown with acoustic stimuli [52, 58, 83, 84]. Latencies, however, were not as short as described with acoustic stimulation [75, 85–87], which may be due to differences not only in afferent conduction times but also in stimulus characteristics as described above.

Exploring startle responses, Ravichandran et al. [34, 88] reported large EMG activity in arm muscles when applying elbow disturbance to the same arm, thus making it impossible to ascertain whether a given response was part of a LLR or of an advanced voluntary reaction. Additionally, the authors used only one level of velocity as disturbance and did not specify whether it was mean or peak velocity. Different kinematic stimuli have previously been used in RT trials [34, 43–45, 56, 61, 89], partly in combination with other stimulus modalities. The authors described the applied stimuli in different dimensions (velocity, acceleration…) and values (peak value, mean value…) and explored various motor reactions, which may have required different periods of time either to perceive the stimulus, or to execute the motor response, thus explaining at least in part different results among these studies and the present one. Notably, in a recent study Campbell et al. [43] reported RTs of less than 100 ms following balance perturbation. In the present study RTs were substantially longer, because we intentionally wanted to avoid intersensory facilitation with vestibular afferents by suspending the subjects in the Lokomat system. Furthermore, our kinematic perturbation was rather long-lasting, yet being able to elicit startle signs and a StartReact effect in most trials at 240°/s passive left and right KF (Figure 2). The low range of StartReact effects in the present study concurs with other startle paradigms and stimulus modalities [41, 51, 69, 70, 85, 90, 91]. Notably, ECR responses appeared later than preprogrammed upper limb postural responses to lower limb perturbations [92]. They appeared, however, in the early time range of voluntary responses reported to occur around 250 ms [93–95]. Finally, more attention directed to stimulus detection in one limb rather than to the RT task in the other limb may have contributed to response latencies, in particular for low stimulus intensities, an effect previously described as “cost in responses due to attentional shifting” [96].

Trials at 240°/s without startle signs had shorter latencies for WE than trials at 6°/s or 60°/s but had longer latencies than those trials at 240°/s containing startle signs. These results suggest, in addition to a progressive influence of stimulus intensity on voluntary movement responses, an additional superimposed startle effect, both being independent of each other [41]. Notably, in the auditory domain, the presence of startle signs depends not only on stimulus intensity [41], but also on the degree of the subject's preparedness for a task [74, 75].

Furthermore, when subjects are informed about the upcoming stimulus, the probability of ensuing startles is reduced. Accordingly, in situations when subjects knew in advance both side and velocity of leg movement (240Known), no startles occurred in spite of the subjects' high level of preparedness to react with WE, as evidenced by response latencies which were equally fast in conditions 240Known and 240React. In conditions 240React and 240StartReact, we assumed that subjects expected that the leg which was usually displaced was also to be moved next. However, subjects were uncertain of the velocity, which was usually low (6°/s and 60°/s), and indeed we found only few trials with StartReact effects at low velocities. Most pronounced StartReact effects with shortest latencies and largest EMG bursts in ECR were observed when the movement occurred in the contralateral leg, that is, least expected. This phenomenon of response latency shortening related to a subject's expectancy of the stimulus has previously been described [97, 98]. It pertains to a higher perceptual arousal for selected stimuli, in this case, stimulus location. Therefore, a prepared response can be modulated by the degree of knowledge of the characteristics of the upcoming stimulus (expectancy). A startle reflex may occur when a subject is confronted with a certain stimulus novelty (e.g., high intensity). However, when a second stimulus novelty is added (e.g., contralateral leg), the resulting response is further facilitated in form of an augmented startle reflex and a StartReact effect.

### 4.4. Limitations of the Study

Time was the most critical limiting factor in the present study, and a compromise had to be achieved between number and type of trials to be executed according to study protocol and the subjects' ability to cope with being suspended in the Lokomat, continuous attention, and increasing fatigue. The main goal was to unequivocally document the presence of startle signs and a StartReact effect in response to kinematic stimuli, while excluding vestibular influences, separating reflexive from voluntary reactions, and differentiating startle signs from muscle activity inherent to postural or voluntary motor patterns. As shortening in RT may be due to a pure intensity effect and as startle signs were present only in few trials, additional trials had to be appended on-line in order to achieve the minimum required number of trials with startle signs. However, each of the 240°/s trials had to be interspersed with at least 5 trials at 6°/s and occasional trials at 60°/s, in order to avoid rapid habituation and to maintain unexpectedness of high intensity stimuli. Adding trials with startling acoustic stimuli as IS for further comparison with previous studies was thus not feasible. Furthermore, additional trials with prepulses were not possible for the same reason, but those included served as additional means to corroborate the StartReact effect even in the absence of an overt startle reflex.

In summary, our results provide evidence that a kinematic stimulus is able to elicit a startle reflex and a StartReact effect. A vestibular route does not seem to be required nor intersensory facilitation. The responses seem to depend on preparedness and expectancy of the subject, as well as the intensity of the stimulus over time and its detection by the subject. These findings have important implications in neurorehabilitation. Interest in quantification of spastic muscle tone by means of robotic devices has recently increased. When measuring muscle stiffness in the Lokomat [13], unwanted “contamination” by startle responses could be avoided either by implementing prepulse inhibition, or by excluding the first one or two recordings (first trial effect). On the other hand, desired startle responses can help patients performing movements which are otherwise impossible to being executed due to neural lesions affecting the corticospinal tract. Finally, novel therapeutic strategies can be envisioned implementing startle reflexes in the training of compensatory reactions in people, who are exposed to unstable or slippery surfaces, in particular workers, elderly, or handicapped people, in order to prevent falls.

## Figures and Tables

**Table 1:** Experimental protocol applied to each subject. Except for the first block of trials (WE-only), the sequence of all other trials was pseudorandomized. After performing each single trial, resulting recordings were categorized according to the presence or absence of startle reflex signs into distinct conditions. The column *number of trials* includes the sum of both respective conditions (with and without startle reflex signs).

Velocity (°/s)	Conditions	Number of trials	Prepulse	Imperative signal	Wrist extension
—	WE-only	5 trials	−	Sound	+
6	6React	≥30 trials	−	KF	+
	6StartReact				
60	60React	≥10 trials	−	KF	+
	60StartReact				
240	240React	≥5 trials	−	KF	+
	240StartReact				
240	240PrepReact	≥2 trials	+	KF	+
	240PrepStartReact				
240	240Control	≥2 trials	−	KF	−
	240StartControl				
240	240ContraReact	≥4 trials	−	Contralateral KF	+
	240ContraStartReact				
240	240Known	5 trials	−	KF	+
	240StartKnown				

## Abbreviations

ECR: Extensor carpi radialis muscle
EMG: Electromyography
iEMG: Integrated EMG area-under-the-curve
IS: Imperative signal
KF: Knee flexion
LLR: Long loop reflex
OOc: Orbicularis oculi muscle
RT: Reaction time
SCM: Sternocleidomastoid muscle
WE: Wrist extension.
